# Risk prediction models for depression in patients with coronary heart disease: a systematic review and meta-analysis

**DOI:** 10.3389/fcvm.2024.1522619

**Published:** 2025-01-15

**Authors:** Jie Zhang, Yue Zhou, Linyu Huang, Xingling Zhang, Long Li, Chongcheng Xi

**Affiliations:** ^1^ School of Nursing, Chengdu University of Traditional Chinese Medicine, Chengdu, China; ^2^ School of Basic Medicine, Chengdu University of Traditional Chinese Medicine, Chengdu, China

**Keywords:** coronary heart disease, depression, prediction models, systematic review, meta-analysis

## Abstract

**Background:**

Risk prediction models for depression in patients with coronary heart disease are increasingly being developed. However, the quality and applicability of these models in clinical practice remain uncertain.

**Objective:**

To systematically evaluate depression risk prediction models in patients with coronary heart disease (CHD).

**Methods:**

Databases including PubMed, Web of Science, Embase, Cochrane Library, CNKI, Wanfang, VIP, and SinoMed were searched for relevant studies from inception to September 29, 2024. Two researchers independently screened the literature, extracted data, and used the Prediction Model Risk of Bias Assessment Tool (PROBAST) to evaluate the models' risk of bias and applicability.

**Results:**

Eight studies, encompassing 13 risk prediction models and involving 8,035 CHD patients, were included, with 1,971 patients diagnosed with depression. Common predictors included age, educational level, gender, and cardiac function classification. The area under the curve (AUC) for the models ranged from 0.772 to 0.961, indicating overall good performance; however, risk of bias was high, primarily due to issues in the analysis phase, such as inadequate handling of missing values, univariate analysis for variable selection, and lack of external validation.

**Conclusion:**

Depression risk prediction models for CHD patients generally perform well, but high risk of bias and limited applicability remain concerns. Future studies should focus on developing and validating more robust models to aid healthcare professionals in early identification of high-risk patients for depression.

**Systematic Review Registration:**

https://www.crd.york.ac.uk/prospero/display_record.php?ID=CRD42024625641, identifier (CRD42024625641).

## 1 Introduction

Coronary Heart Disease (CHD) is an ischemic heart disease caused by coronary artery atherosclerosis, leading to vessel lumen narrowing or occlusion, which consequently triggers myocardial ischemia, hypoxia, or necrosis (1). According to the World Health Organization (WHO), approximately 9 million people die from CHD each year, making it one of the leading causes of mortality worldwide (2, 3). Clinically, CHD is often treated with Percutaneous Coronary Intervention (PCI), a procedure that rapidly restores coronary blood flow and significantly reduces mortality rates (4). However, CHD is marked by a high recurrence rate, particularly among patients who have experienced myocardial infarction, as they often develop depressive symptoms from recalling near-death experiences. Studies indicate that the prevalence of depression among CHD patients ranges from 20% to 50%, considerably higher than in the general population (5). Depression is not only an independent risk factor for CHD onset but also a key predictor of poor prognosis (6). Research has shown that depression increases the risk of myocardial infarction recurrence by 1.3 times and elevates mortality risk by 1.8 to 2 times (7). These findings underscore the profound impact of depression on CHD patients, as it not only exacerbates the condition but also markedly increases mortality. Therefore, selecting or developing scientifically validated risk prediction models to identify high-risk patients early is crucial. Although several predictive models for depression in CHD patients have been developed (8, 9), their quality and predictive performance vary significantly, and a systematic review is lacking. This study aims to comprehensively review depression risk prediction models in CHD patients, systematically evaluate their bias and applicability, provide clinical guidance for selecting reliable models, and inform future model improvement and development.

## 2 Methods

The protocol for this systematic review and meta-analysis is available in the PROSPERO database (CRD42024625641).

### 2.1 Inclusion and exclusion criteria

#### 2.1.1 Inclusion criteria

(1) P (Population): Age ≥18 years, diagnosed with coronary heart disease (CHD) according to established criteria; (2) I (Intervention model): Development or validation of a depression risk prediction model for CHD patients, including predictors ≥ 2; (3) C (Comparator): No comparator model; (4) O (Outcome): The primary outcome was depression.

#### 2.1.2 Exclusion criteria

(1) Articles without full-text availability; (2) Duplicate publications; (3) Conference abstracts and dissertations; (4) Non-Chinese or non-English publications.

### 2.2 Search strategy

A computer-based search was conducted across four English databases (Web of Science, PubMed, Embase, and Cochrane Library) and four Chinese databases (CNKI, Wanfang, VIP, and SinoMed) to identify studies related to depression risk prediction models in CHD patients. The search period spanned from database inception to September 29, 2024. A combination of subject terms, free terms, and Boolean operators was used for both Chinese and English searches. Chinese search terms included coronary heart disease, acute coronary syndrome, myocardial infarction, post-PCI, depression, depressive state, prediction, predictive factors, influencing factors, risk assessment, model, and tool. English search terms included coronary disease, acute coronary syndrome, myocardial infarction, post-PCI, depression, depressive disorder, prediction, predictors, influencing factors, risk assessment, model, tool, and score. For each database, a tailored search strategy was developed based on its unique features. Additionally, references in included studies were reviewed to identify supplementary relevant literature.

### 2.3 Literature screening and data extraction

Two researchers independently screened the literature and extracted data based on the inclusion and exclusion criteria, followed by cross-checking the data results. Any disagreements were resolved through discussion or decided by a third researcher. The extracted data included the following details: first author, year, country, study type, study population, depression diagnostic criteria, sample size, modeling method, area under the receiver operating characteristic curve (AUC), and model presentation format.

### 2.4 Risk of bias and applicability assessment

The Prediction model Risk Of Bias ASsessment Tool (PROBAST) (10) was used to assess the risk of bias and applicability of the included models. The risk of bias evaluation covers four domains: participants, predictors, outcomes, and analysis, with a total of 20 specific questions. Based on the “shortcoming theory,” each domain's results were synthesized as follows: if all items are marked as “probably yes” or “yes,” the domain is rated as “low risk”; if any item is rated as “no” or “probably no,” the domain is deemed “high risk”; if an item lacks sufficient information, the domain is rated as “unclear.” For the overall risk of bias, a “low risk” is assigned only when all four domains are rated as “low risk”; if any domain is rated as “high risk,” the overall risk of bias is rated as “high risk”; if any domain is rated as “unclear,” the overall risk of bias is categorized as “unclear.” The applicability assessment includes three domains: study population, predictors, and outcomes, using the same evaluation approach as the risk of bias assessment.

### 2.5 Statistical analysis

Stata 17.0 software was used for quantitative analysis of the AUCs of the included models. Cochrane's Q test was applied to assess heterogeneity among the models, with *I^2^* used to quantify the degree of heterogeneity. If *P* > 0.05 and *I*^2^ ≤ 50%, it indicates no significant heterogeneity among studies, and a fixed-effects model is applied. Conversely, if *P* ≤ 0.05 or *I*^2^ > 50%, it suggests substantial heterogeneity. In such cases, subgroup analysis is conducted to explore the sources of heterogeneity, and sensitivity analysis is performed by sequentially excluding individual studies. If heterogeneity persists, a random-effects model is used for the analysis. To detect potential publication bias, Egger's test was performed, with *P* > 0.05 suggesting a low likelihood of publication bias.

## 3 Results

### 3.1 Study selection

**Figure 1:**
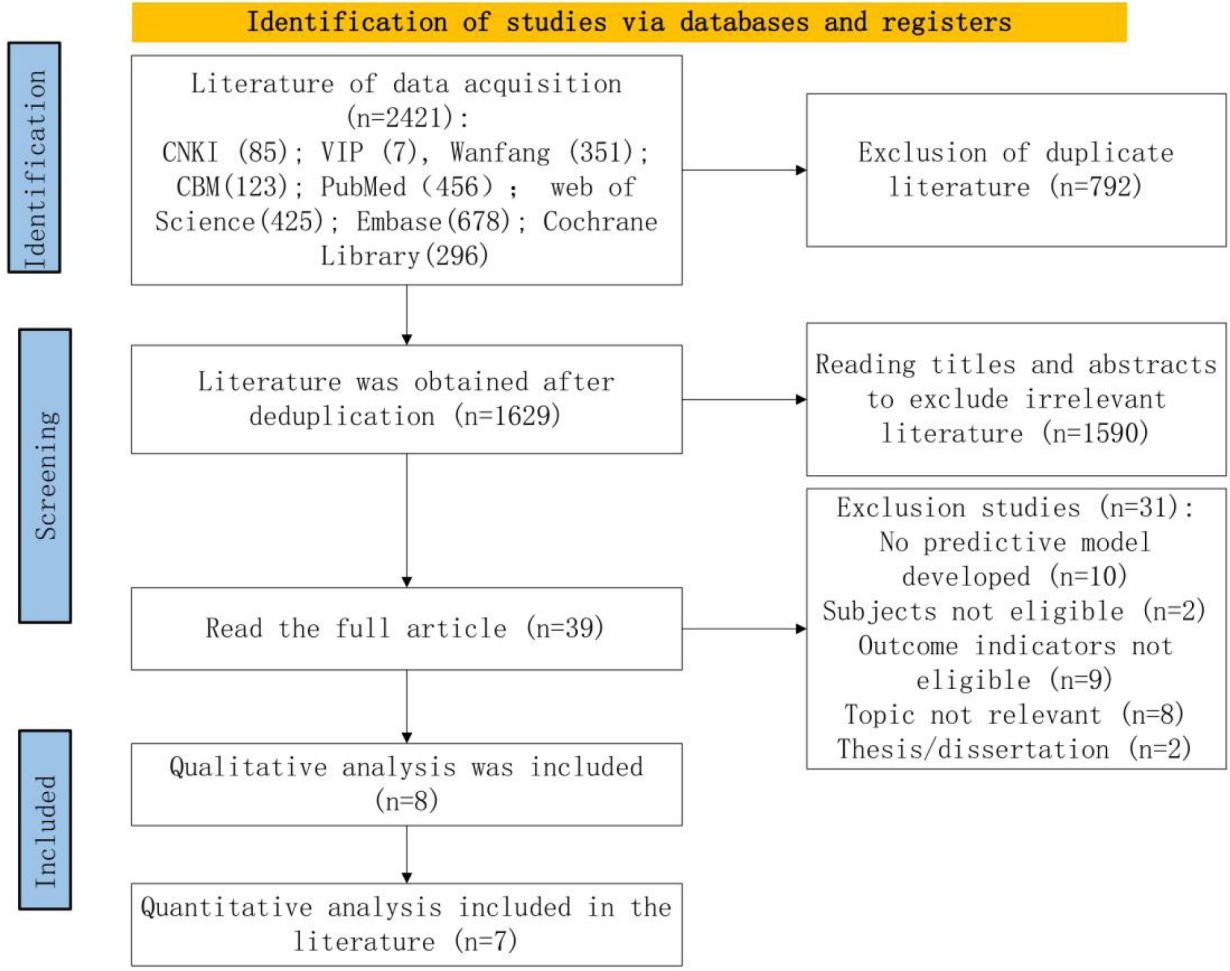
Preferred reporting items for systematic reviews and meta-analysis-conforming flowchart of the screening process.

The initial search yielded 2,421 relevant articles. After removing 792 duplicates, a further 1,590 articles were excluded based on title and abstract screening for topic relevance. Full texts of 39 articles were reviewed, and ultimately, 8 articles were included. The screening process is shown in Figure 1.

### 3.2 Study characteristics

This study included a total of 8 studies (8, 9, 11–16), comprising 3 prospective cohort studies (8, 12, 14), 4 retrospective studies (9, 13, 15, 16) (including 2 retrospective cohort studies (13, 16) and 2 retrospective case-control studies (9, 15), with one being a nested case-control study (15), and 1 cross-sectional study (11). Data for 2 studies (9, 11) were sourced from the U.S. Centers for Disease Control and Prevention's National Health and Nutrition Examination Survey (NHANES), while data for the remaining 6 studies (8, 12–16) were drawn from clinical databases. A total of 8,035 CHD patients were included in the analysis. Details are provided in Table 1.

**Table 1:** Basic characteristics of included studies.

Authors year	Region	Study design	Study population	Depression diagnostic scale	Sample size		
					Total number	Event number	Incidence (%)
Miao (8) 2023	China, Fujian	Prospective Cohort study	Post-PCI	PHQ-9	150	82	54.7
Wang (9) 2022	USA	Retrospective Case-control	Myocardial infarction	PHQ-9	1,615	276	17.1
Hou (11) 2024	USA	Cross-sectional study	CHD	PHQ-9	2,482	401	16.2
Dai (12) 2024	China, Zhengzhou	Prospective Cohort study	In-Stent Restenosis CHD	SDS	252	118	46.8
Zhu (13) 2023	China, Zhejiang	Retrospective Cohort study	Male Post-PCI	PHQ-9	132	40	30.3
Li (14) 2023	China, Hubei	Prospective Cohort study	Post-PCI	HAMD	235	56	23.8
Chen (15) 2019	China, Beijing	Nested case-control study	Post-PCI	HAMD	3,048	967	31.7
Wang (16) 2021	China, Hainan	Retrospective Cohort study	Post-PCI	HAMD	121	31	25.6

PHQ-9, Patient Health Questionnaire-9; SDS, Self-Rating Depression Scale; HAMD, Hamilton Depression Rating Scale.

### 3.3 Basic characteristics of prediction models

**Figure 2:**
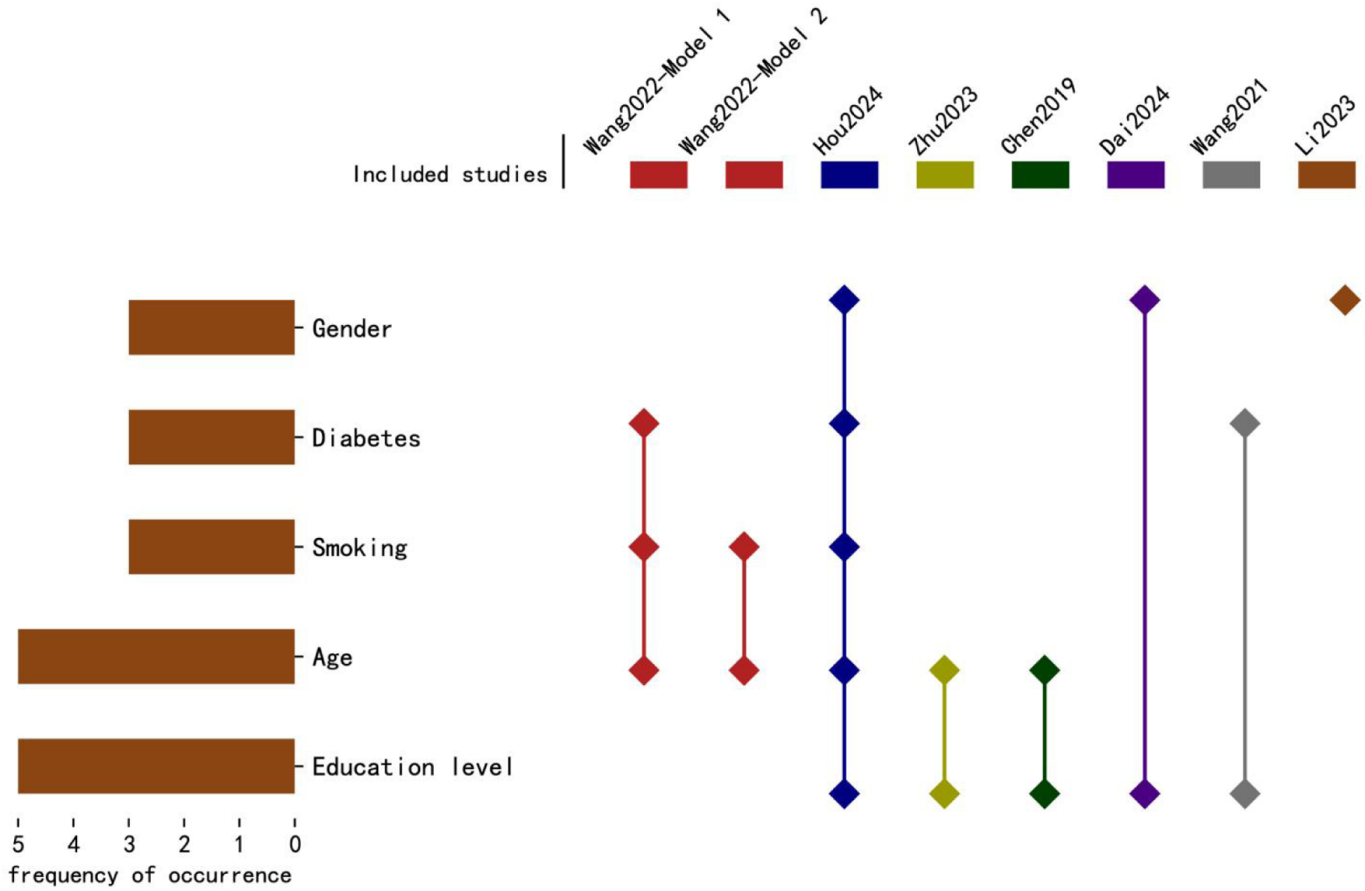
The frequency and distribution of predictive factors in each predictive. The horizontal axis represents the frequency of occurrence of predictive factors, while the vertical axis lists the top five predictive factors. The horizontal bars indicate the frequency of occurrence, and the diamond markers show the distribution of these factors in each study, corresponding to the study names listed above.

The 8 studies constructed a total of 13 prediction models. Hou et al. developed 5 models and ultimately selected an optimal model for nomogram construction, while Wang et al. developed 2 models; each of the remaining studies constructed 1 model. In terms of variable selection, Hou et al.'s optimal model used the best subset selection method, Li Cexing et al. applied Lasso regression, and the other 6 studies utilized univariate analysis to select variables. Regarding modeling methods, Wang et al. employed Lasso regression in their second model, while all other models used logistic regression. In terms of model performance, 8 models reported the area under the curve (AUC), ranging from 0.772 to 0.961; 1 model reported the C-index. Calibration was assessed in 7 studies through calibration plots, 1 study reported the Brier score, and 3 studies conducted the Hosmer-Lemeshow test (Figure 2). Details are provided in Table 2.

**Table 2:** Construction methods and performance of included predictive models.

Authors year	Number of candidate variables	Variable selection	Modeling method	Model performance	
				AUC	Calibration method
Miao (8) 2023	24	Univariate analysis	Logistic	0.857	Calibration Curve + BrierScore = 0.15
Wang (9) 22022	14	Univariate analysis	Model 1:Logistic Model 2:Lasso	Model 1: Development Cohort (0.799) Validation Cohort (0.731) Model 2: Development Cohort (0.772) Validation Cohort (0.771)	Calibration Curve +H-L Test (Development Cohort*P* = 0.449 Validation Cohort*P* = 0.765)
Hou (11) 2024	27	Optimal model: Optimal subset method	Logistic	Training Set: 0.774 Validation Set: 0.72	Calibration Curve
Dai (12) 2024	9	Univariate analysis	Logistic	0.883	Calibration Curve + H-L Test (*P* = 0.11)
Zhu (13) 2023	10	Univariate analysis	Logistic	0.834	Calibration Curve
Li (14) 22023	16	Lasso	Logistic	0.881	Calibration Curve + H-L Test (*P* = 0.425)
Chen (15) 2019	14	Univariate analysis	Logistic	0.961	Not Mentioned
Wang (16) 2021	15	Univariate analysis	Logistic	C = 0.821	Calibration Curve

**Figure 3:**
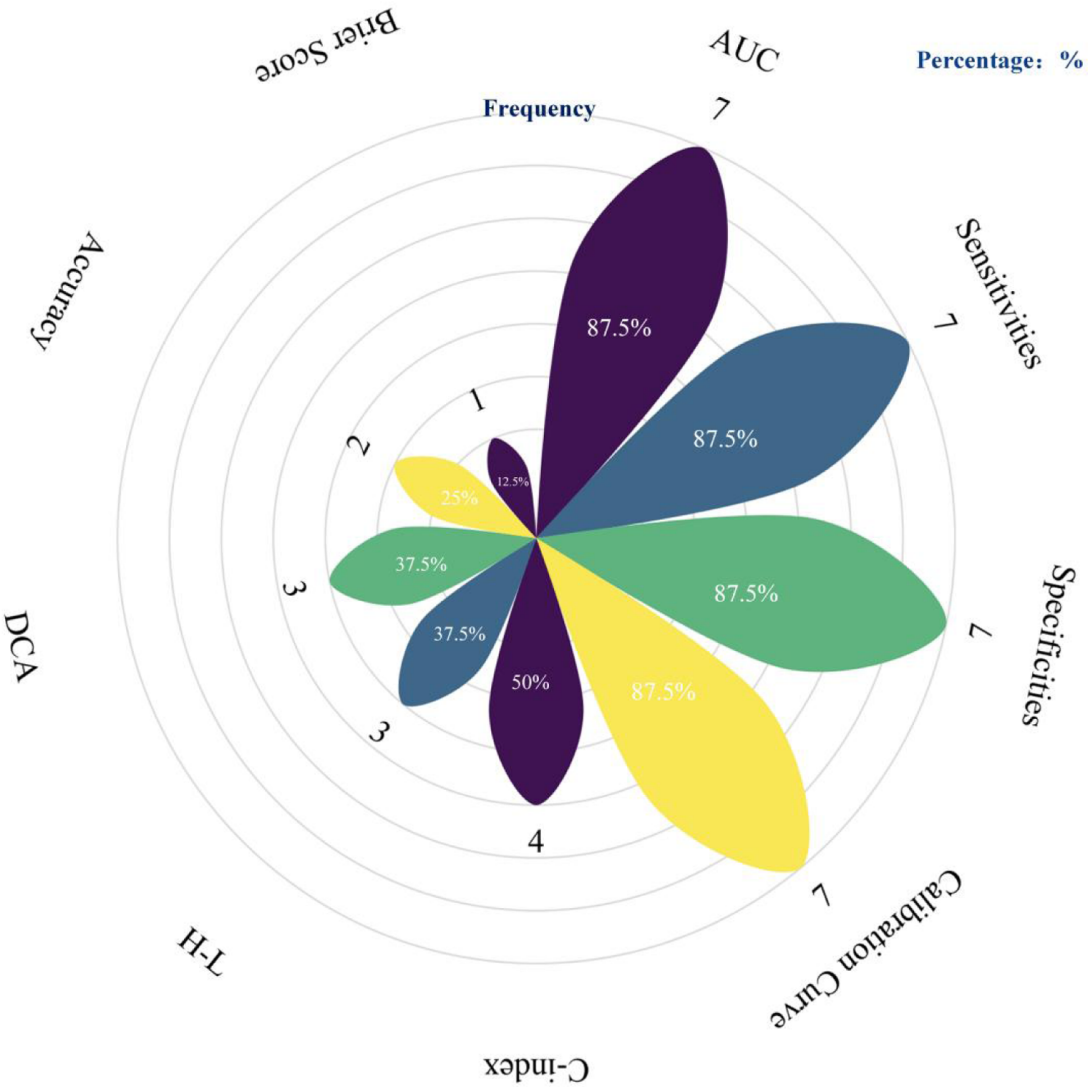
Summary of performance evaluation metrics used in predictive models. The petals represent different performance evaluation metrics, and the length of each petal along with the labeled percentage indicates the frequency of use for the corresponding metric (created using the following website: https://www.chiplot.online/).

For model validation, only 2 studies performed external validation, while 5 conducted internal validation. Regarding model presentation, 7 studies used nomograms, and 1 used regression equations. The number of candidate predictors ranged from 9 to 27, with the final number of included predictors between 3 and 10 (distribution of the top five predictive factors, Figure 3). Additional details are available in Table 3.

**Table 3:** Validation methods and final predictors of included models.

Authors year	Validation method	Model presentation	Final predictors
Miao (8) 2023	Bootstrap internal validation	Nomogram	3: Major life events, post-operative GAD-7 score, baseline PHQ score
Wang (9) 2022	External validation	Nomogram	Model 1 (8): Age, BMI, diabetes, alcohol consumption, smoking, insomnia, exercise, PIR Model 2 (4): Age, smoking, insomnia, PIR
Hou (11) 2024	External validation	Nomogram	10: Age, Gender, Education Level, Marital Status, Hypertension Medication Use, Smoking, Diabetes, HDL-C, AST, Creatinine
Dai (12) 2024	Bootstrap internal Validation	Nomogram	5: Gender, education level, marital status, number of stents, Mehran classification
Zhu (13) 2023	Bootstrap internal validation	Nomogram	4: Education level, age, cardiac function classification, Type A personality
Li (14) 2023	Bootstrap internal validation	Nomogram	5: Female, hypertension, Gensini score, NLR ≥ 3.24, PLR ≥ 147.74
Chen (15) 2019	Not mentioned	Regression Equation	5: Age, Type D personality, cardiac function classification, living alone, education level
Wang (16) 2021	Bootstrap internal validation	Nomogram	5: Education Level, ACS Severity, Hypertension, Diabetes, Lack of Pre-PCI Mental Health Education

GAD-7, Generalized Anxiety Disorder-7; PIR, Poverty-to-Income Ratio; Mehran Classification, Classification of In-Stent Restenosis; Gensini Score, Assessment of Coronary Artery Lesion Severity; NLR, Neutrophil-to-Lymphocyte Ratio; PLR, Platelet-to-Lymphocyte Ratio.

### 3.4 Risk of bias and applicability assessment

#### 3.4.1 Risk of bias assessment

The overall risk of bias in the included studies was relatively high: (1) Participants: Three prospective cohort studies (8, 12, 14) and one nested case-control study (15) were rated as low risk, while four retrospective studies (9, 11, 13, 16) were rated as high risk. The high risk in retrospective studies is primarily due to potential recall bias and the fact that data collection was not initially intended for model development or validation, leading to missing key depression-related predictors in cases, thus increasing bias risk. (2) Predictors: Five studies (12–16) were rated as low risk, while three studies (8, 9, 11) were rated as high risk. Miao's (8) study included the postoperative GAD score as a predictor, which cannot be obtained when using the model, impacting accuracy and resulting in a high risk of bias. Wang's (9) and Hou's (11) studies used self-reported predictors, which increased subjectivity and outcome uncertainty, hence rated as high risk. (3)Outcomes: Two studies (12, 14) were rated as low risk, one study (8) as high risk, and five studies (9, 11, 13, 15, 16) as unclear. Miao's (8) study used baseline PQH scores as predictors, potentially overestimating their association with outcomes, resulting in high risk. The other five studies (9, 11, 13, 15, 16) did not report assessor training, leaving the objectivity of depression outcomes uncertain, hence rated as unclear. (4)Analysis: All included studies were rated as high risk. Three studies (13, 14, 16) had an events-per-variable (EPV) ratio of less than 20, increasing bias risk. One study (12) excluded subjects with missing data directly, leading to a high risk of data bias. Six studies did not mention handling of missing data. Six studies (8, 9, 13–16) relied on univariate analysis for variable selection, which increases bias risk. None of the studies (8, 9, 11–16) addressed complex data processing methods. One study (15) did not report discrimination or calibration and did not account for model overfitting, contributing to a high risk of bias. Details are provided in Table 4.

**Table 4:** Risk of bias and applicability assessment of included studies.

Authors year	Risk of Bias				Applicability			Overall	
	Participants	Predictors	Outcome	Analysis	Participants	Predictors	Outcome	Risk of bias	Applicability
Miao 2023	+	−	−	−	+	−	+	−	−
Wang 2022	−	−	?	−	+	+	+	−	+
Hou 2024	−	−	?	−	+	+	+	−	+
Di 2024	+	+	+	−	−	+	+	−	−
Zhu 2023	−	+	?	−	−	−	−	−	−
Li 2023	+	+	+	−	+	+	+	−	+
Chen 2019	+	+	?	−	+	+	+	−	+
Wang 2021	−	+	?	−	+	+	+	−	+

+: Low risk of bias/High applicability; −: High risk of bias/Low applicability; ?: Unclear.

#### 3.4.2 Applicability assessment

Five studies (9, 11, 14–16)demonstrated generally good applicability, while the remaining three studies (8, 12, 13) showed poor applicability: (1) Participants: Dai Xuehui's (12)study focused on CHD patients with in-stent restenosis, and Zhu Hupei's (13) study limited participants to male subjects, which reduces the applicability of these studies to the general population. (2) Predictors: Miao's (8) study included inappropriate predictors (such as the postoperative GAD score), while Zhu Hupei's (13) study did not report definitions or assessment methods for the predictors, affecting applicability. (3) Outcomes: All studies assessed depression using validated scales; however, Zhu Hupei's (13) study combined anxiety and depression as a single outcome measure without separate analysis, which reduced its applicability. Details are provided in Table 4.

### 3.5 Meta-analysis of included models

**Figure 4:**
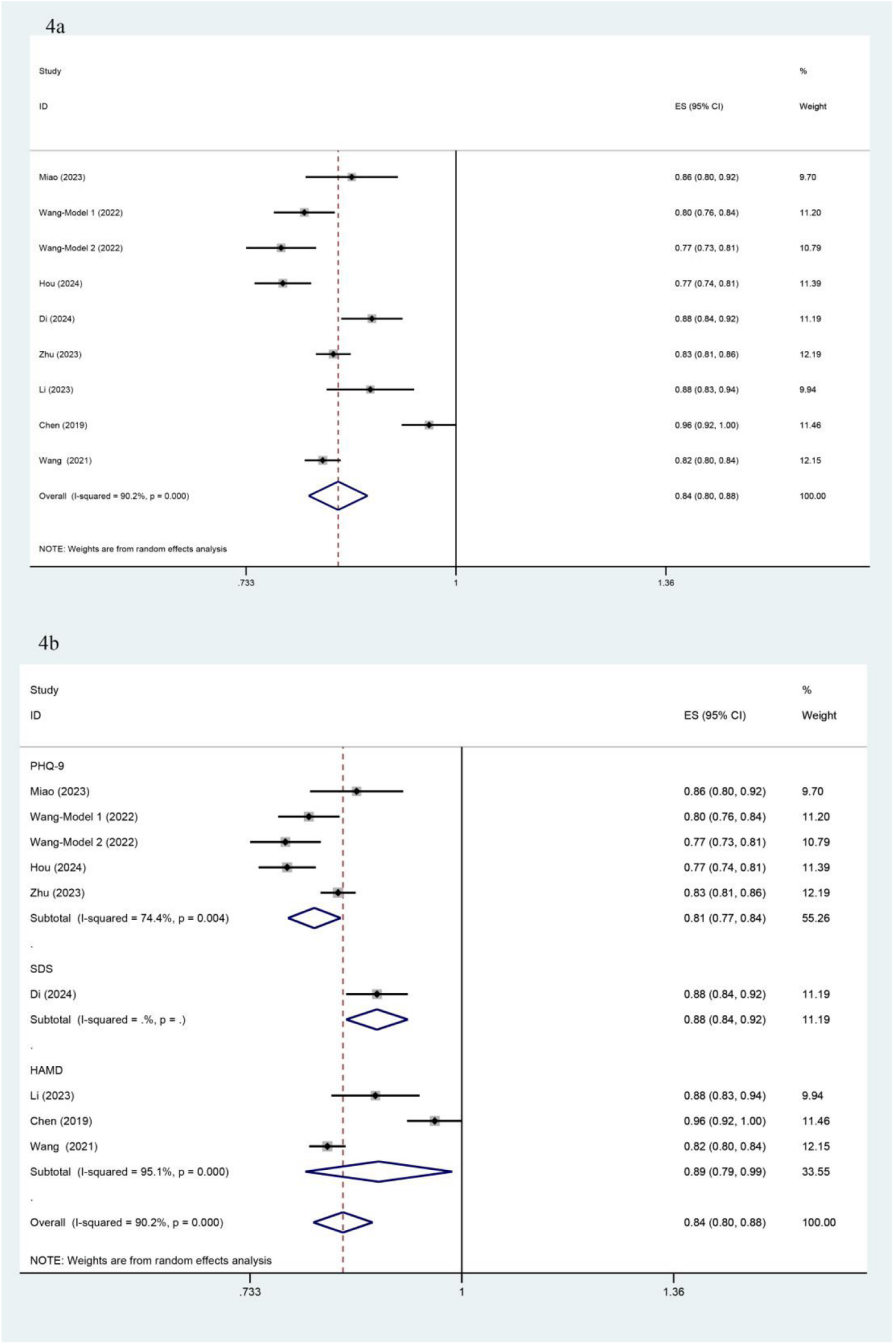
Forest plot and subgroup analysis based on AUC. **(a)** presents the overall forest plot of the 9 included models, based on AUC values and their 95% confidence intervals. (b) shows the forest plot for subgroup analysis by different outcome measurement scales, displaying the AUC values and their 95% confidence intervals within each subgroup.

Among the 8 included studies with a total of 9 models, significant heterogeneity was observed (*I*^2^ = 90.2%, *P* < 0.001), prompting the use of a random-effects model for analysis. The pooled AUC estimate was 0.84 (95% CI: 0.80–0.88), indicating good overall predictive performance (Figure 4a). The substantial heterogeneity among studies may be attributed to differences in study design, patient characteristics, and outcome evaluation metrics. Subgroup analysis based on different outcome measurement scales showed that heterogeneity was *I*^2^ = 74.4% for the PHQ-9 group and *I*^2^ = 95.1% for the HAMD group, with no significant reduction in heterogeneity, suggesting that measurement tools were not the primary source of heterogeneity (Figure 4b). Sensitivity analysis, conducted by sequentially excluding individual studies, revealed no significant changes in heterogeneity, further supporting the robustness of the findings. Egger's test yielded a *P*-value of 0.767 (*P* > 0.05), indicating no significant publication bias.

## 4 Discussion

### 4.1 Model performance is good but bias risk is high; focus needed on external validation and diverse modeling

The commonly used metrics for evaluating model performance include AUC and calibration. In this study, all 9 included models demonstrated AUCs above 0.7, with 6 models reaching or exceeding 0.8, indicating good discriminative ability. Additionally, the calibration curves for 8 models closely aligned with the diagonal line, suggesting strong agreement between predicted probabilities and actual occurrence rates. Four models also underwent Hosmer-Lemeshow (H-L) testing, with *P*-values ≥0.05, further supporting good calibration. Overall, these models effectively identify high-risk patients for depression, showing favorable performance; however, the high risk of bias persists.

First, there is bias in the data sources. (1) Most data are from single-center studies with insufficient sample sizes, and five studies are retrospective analyses, introducing recall bias to some extent and affecting the model's quality. (2) Depression diagnosis is highly subjective and requires professional assessment; however, five studies did not mention the training of evaluators, which may lead to outcome bias. Future studies should prioritize multicenter, large-sample, high-quality prospective studies to minimize recall bias. Additionally, outcome evaluators should be uniformly trained independent third parties to enhance the consistency and accuracy of depression diagnoses.

Second, biases also exist in model construction. (1) In terms of variable selection, six studies relied on univariate analysis to identify predictors, potentially omitting important factors and leading to model overfitting, which weakens predictive power (17). (2) Six studies did not report missing values, and one study directly excluded missing data. This approach may introduce bias in the associations between predictors and outcomes, and even in the absence of bias, missing data can reduce precision, widening confidence intervals. (3) Six studies used traditional logistic regression to build models, limiting the ability to capture complex relationships among variables, which affects model accuracy and stability (18). Therefore, future research should combine domain knowledge and clinical experience and cautiously select variables using methods like LASSO regression and stepwise regression (19). When handling missing data, methods such as multiple imputation and single imputation should be used to mitigate the adverse effects of missing data on statistical analysis and model stability (20). Additionally, incorporating machine learning and deep learning approaches can enhance the accuracy and adaptability of predictive models (21).

Finally, there are some limitations in model validation. The predictive performance of a model can be affected by variations in populations and regions, underscoring the need for thorough validation during model development. Internal validation assesses model reproducibility and prevents overfitting, while external validation evaluates transferability and generalizability, regarded as the “gold standard” of validation (22, 23). In this study, three models underwent external validation but lacked internal validation, potentially impacting model performance and reliability; five models performed internal validation without external validation, with study populations mainly composed of Chinese individuals, limiting the model's generalizability and applicability. Future research should emphasize external validation, particularly across diverse regions, ethnicities, cultural backgrounds, and lifestyle factors, to enhance model generalizability. Additionally, variations in coronary heart disease types (e.g., stable angina, acute coronary syndrome, post-PCI), as well as different disease stages, should be considered. Treatment modalities and levels of social support may also influence predictive performance. Taking these factors into account comprehensively will contribute to improving model reliability and applicability.

### 4.2 Predictor differences and commonalities: focus on age, education level, and gender

The nine models in this study included between 3 and 10 predictors, primarily categorized into four groups: demographic factors (e.g., age, gender), psychological factors (e.g., PHQ score, personality), clinical factors (e.g., hypertension, number of stents), and lifestyle factors (e.g., smoking, alcohol consumption). Despite variations in predictor selection due to study types and included variables, some commonalities were identified. Predictors frequently appearing across eight studies included age, education level, gender, and cardiac function classification. Wang's study (9) found a negative correlation between age and depression, a finding supported by Murphy et al. (24). Conversely, Zhu Hupei (13) and Chen Hongyu (15) indicated that age over 60 is an independent risk factor for post-PCI depression. This may be because younger patients feel disoriented by sudden illness, while older patients, experiencing functional decline, may perceive themselves as a burden. Research (12) suggests that patients with higher education levels have stronger comprehension and application abilities when receiving health education, resulting in better prognosis and lower risk of depression. Multiple studies (25–27) found that females are more prone to depression, likely due to hormonal fluctuations during menstruation, menopause, and perinatal periods, which contribute to emotional instability. Additionally, women often bear more responsibilities and pressures in social and family roles, increasing depression risk. Higher cardiac function classifications are associated with more pronounced symptoms of dyspnea and chest tightness, as well as greater limitations in daily life and physical activity. Such physiological discomfort amplifies psychological stress, eroding confidence in life, and leading to negative emotions or even self-harm and suicidal behaviors (28). Thus, early screening should focus on these common factors to promptly identify high-risk individuals. However, this study also found that many current predictors are challenging to directly intervene upon, limiting nursing interventions. Future research should consider including modifiable factors, such as sleep quality, psychological state, and medication adherence, to enable more targeted nursing interventions and enhance clinical outcomes.

### 4.3 Limitations of this study

(1) Seventy-five percent (6/8) of the studies included were based on data from China, which may introduce regional bias and limit the applicability of the findings to Western populations. (2) Seventy-five percent (6/8) of the studies did not perform external validation, restricting the generalizability of the models. (3) The included studies were limited to those published in Chinese and English, potentially introducing language bias and failing to capture findings from studies published in other languages.

## 5 Conclusion

The predictive models included in this study demonstrated generally good performance; however, as evaluated by PROBAST, the overall risk of bias remains high, and the models' applicability needs improvement. Currently, risk prediction models for depression in patients with coronary heart disease do not yet meet established standards. Future researchers should develop and validate more scientifically robust risk prediction models in accordance with PROBAST guidelines.

## Data Availability

The original contributions presented in the study are included in the article/Supplementary Material, further inquiries can be directed to the corresponding author.
