# Supplementary material for: Risk prediction models for depression in patients with coronary heart disease: a systematic review and meta-analysis
Source: Front Cardiovasc Med. 2025 Jan 15;11:1522619. doi: 10.3389/fcvm.2024.1522619 (PMC11774958; doi:10.3389/fcvm.2024.1522619)

S1

PubMed search strategy

#1 (((Acute Coronary Syndrome[MeSH Terms]) OR (Coronary Disease[MeSH Terms])) OR (Myocardial Infarction[MeSH Terms])) OR ("Coronary Disease"[Title/Abstract] OR "Coronary Heart Disease"[Title/Abstract] OR "Disease, Coronary"[Title/Abstract] OR "Angina Pectoris"[Title/Abstract] OR "Myocardial Infarction"[Title/Abstract] OR "acute coronary syndrome"[Title/Abstract] OR "post-PCI"[Title/Abstract])

#2 (Depression[MeSH Terms]) OR (Depression[Title/Abstract] OR Emotional Depression[Title/Abstract] OR Depressive Disorder[Title/Abstract])

#3 ("risk assessment"[Title/Abstract] OR "risk prediction"[Title/Abstract] OR "predict"[Title/Abstract] OR "predicts"[Title/Abstract] OR "prognosis"[Title/Abstract] OR "forecast"[Title/Abstract])

#4 ("model"[Title/Abstract] OR "tool"[Title/Abstract] OR "score"[Title/Abstract])

#5 #1 AND #2 AND #3

S2

Sensitivity analysis of the eight included studies

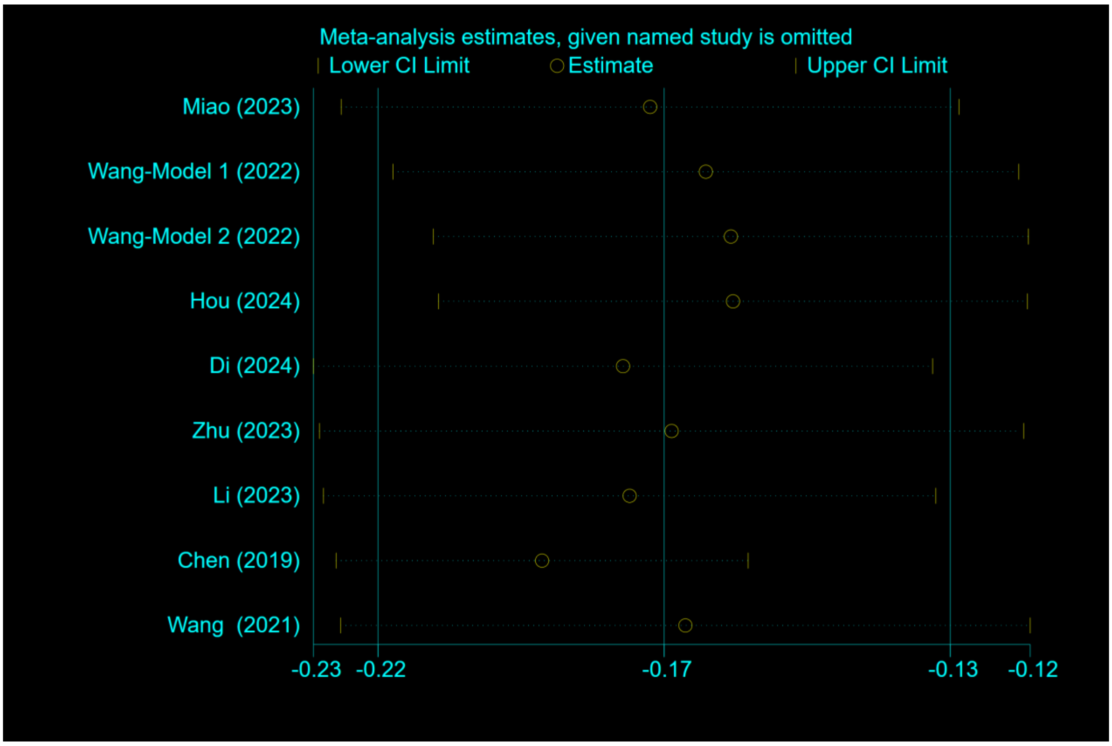

Supplement: Supplementary file 1 [file Datasheet1.pdf]
